# Development of Laser-Scribed Graphene Electrodes for Label-Free L-Histidine Sensing in Artificial Sweat

**DOI:** 10.3390/bios16060318

**Published:** 2026-06-02

**Authors:** William García-Rodríguez, Karla Echeverría-Altamar, José A. Lasalde-Ramirez, Pedro J. Resto-Irizarry

**Affiliations:** 1 Department of Mechanical Engineering, University of Puerto Rico, Mayagüez Campus, Mayagüez, PR 00680, USApedroj.resto@upr.edu (P.J.R.-I.); 2 Bioengineering Graduate Program, University of Puerto Rico, Mayagüez Campus, Mayagüez, PR 00680, USA

**Keywords:** LSG electrodes, factorial design, central composite design, L-Histidine

## Abstract

This study investigates the fabrication of laser-scribed graphene (LSG) electrodes on polyimide substrates using a CO_2_ laser cutter for label-free L-Histidine detection in artificial sweat. Two-level full factorial and central composite designs were employed to optimize critical manufacturing parameters, including laser speed, power, and electrode width. Electrochemical characterization using cyclic voltammetry with K_3_Fe[CN]_6_ demonstrated superior LSG electrode performance compared to standard glassy carbon electrodes, exhibiting a 702 ± 62% higher oxidation current peak at 0.56 mM K_3_Fe[CN]_6_ in 0.1 M KCl. We successfully demonstrated the label-free electrochemical detection of L-Histidine in artificial sweat using these LSG electrodes. The results show a linear relationship (R^2^ = 0.987) between current peak and L-Histidine concentration within the 8.3 mM to 50 mM range, demonstrating high sensitivity towards L-Histidine. These findings highlight the potential of this optimized LSG electrode fabrication approach for developing high-performance, user-friendly, and disposable wearable biosensors for real-time and non-invasive health monitoring applications in sweat analysis.

## 1. Introduction

Graphene, a 2D monolayer of carbon atoms arranged in a honeycomb lattice, possesses unique electronic properties, including high conductivity and large surface area, making it attractive for electronic applications [1,2]. While early graphene research explored exfoliation [3,4,5] and SiC thermal decomposition [6,7], chemical vapor deposition (CVD) [8] and laser scribing [9,10,11] have emerged as scalable methods. Laser scribing, a process that produces laser-scribed graphene (LSG), offers a compelling alternative for biosensor fabrication due to its low-cost, rapid prototyping, and suitability for flexible substrates, making it particularly attractive for wearable biosensor development [12,13]. Biosensing applications are expanding beyond traditional targets to include neurochemicals, hydrogen peroxide, non-electroactive molecules (using enzyme-based methods), proteins (e.g., cancer biomarkers), immunoglobulins, and viral/bacterial DNA [14,15,16,17,18,19,20]. LSG has demonstrated promise in various biosensing applications, including the detection of biomolecules in complex matrices [14,21,22,23].

However, the electrochemical performance of LSG electrodes is highly dependent on manufacturing parameters. Several studies have investigated the impact of laser scribing parameters (power, speed, geometry) on the structural and morphological properties of LSG [24,25,26,27,28]. A consensus on optimal settings remains elusive, highlighting the need for systematic optimization to maximize the electrochemical signal and sensitivity of the LSG electrodes for analyte detection. Researchers have probed the effect of manufacturing parameters on the structural properties of graphene, including the number of lasing passes, laser focus at different speeds, and power levels. The results highlight problems with laser speed and power control in LSG electrode fabrication, which significantly affect the quality of the produced graphene [26,27]. These results open the door for studying how lasing parameters affect the electrochemical detection properties of LSGs and where the use of design of experiments (DoE) emerges as a valuable technique for efficiently managing measurement resources and time [26,27,28]. Full factorial design (FFD) and central composite design (CCD) are two widely used DoE methods used to study the relative effect of various inputs on a system [29,30]. While laser-scribed graphene is a well-established technique, there remains a need for systematic optimization strategies that link fabrication parameters with electrochemical performance. This work addresses this gap through a combined experimental and analytical framework based on design of experiments (DoE).

In this work, we employ a DoE approach, specifically a 2^3^ full factorial design (FFD) followed by a central composite design (CCD), to efficiently investigate the influence of laser speed, power, and electrode geometry on the electrochemical performance of LSG electrodes, allowing for the selection of the most appropriate manufacturing parameter values. The performance of the selected LSG electrode was then evaluated by comparing it with a standardized glassy carbon (GC) electrode (2 mm OD), with both serving as working electrodes. Electrochemical measurements were conducted using K_3_[Fe(CN)_6_] in KCl as the electrolyte solution, alongside a platinum (Pt) counter electrode and an Ag/AgCl reference electrode. The results demonstrate the potential of LSG as a promising candidate for integration into wearable technology.

Wearable devices show great promise as non-invasive, real-time biosensors for health monitoring by detecting biomarkers in body fluids such as urine, saliva, and sweat [14,18,22,31,32,33]. Sweat is a highly viable option for real-time monitoring of a wide range of analytes, including amino acids like L-Histidine [34,35,36,37,38,39]. Furthermore, label-free electrochemical detection is particularly advantageous for wearable sensing as it requires minimal sample preparation and enables continuous monitoring. L-Histidine, a precursor to histamine, a key mediator in allergic reactions, is a promising target for non-invasive, continuous monitoring of allergic responses using wearable biosensors. Its presence in sweat makes it particularly attractive for wearable biosensor applications. Studies report L-Histidine concentrations in human sweat ranging from 0.212 mM to 3.3 mM [36,37]. To evaluate the detection capability of the fabricated LSG electrode as a label-free sensor, a commercially available artificial sweat solution [38], consisting of 0.5% NaCl, 0.05% L-Histidine, and 0.50% Na_2_HPO_4_, was used. This standard formulation was chosen because it mimics the major inorganic components of human sweat, providing a controlled and reproducible matrix for electrochemical measurements. This study evaluates the LSG electrode’s performance across an L-Histidine concentration range from 3.2 mM to 50 mM, encompassing and extending beyond the reported physiological range, allowing for a comprehensive characterization of the sensor’s performance. We anticipate that this optimized LSG electrode fabrication approach will enable the development of high-performance, user-friendly, and disposable wearable biosensors for real-time, non-invasive health monitoring applications in sweat analysis.

## 2. Materials and Methods

**Figure 1:**
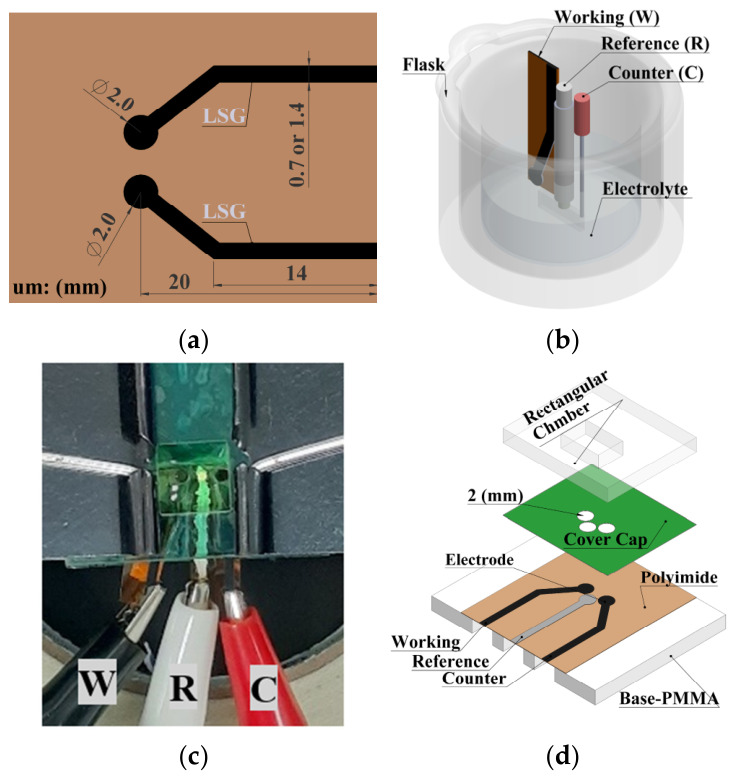
Experimental setup used for electrode characterization and testing. (**a**) Geometry for manufacturing laser-scribed electrodes. (**b**) The first experimental setup utilizes an LSG as the working electrode, Ag/AgCl as the reference electrode, and Pt as the counter electrode. All three electrodes are connected to a potentiostat and submerged in an electrolyte solution of K_3_[Fe(CN)_6_] in 0.1 M KCl within a glass flask. (**c**) The second experimental setup employs two LSG electrodes as the working and counter electrodes, along with a reference electrode prepared using silver ink. (**d**) Exploded geometry provides a detailed view of the second experimental setup. This configuration features LSG and silver electrodes fabricated on a polyimide substrate, mounted on a polymethyl methacrylate (PMMA) film, and covered with a vinyl layer to confine contact between the electrodes and the electrolyte. Additionally, a rectangular PMMA chamber is incorporated on the surface to control the volume of the deposited electrolyte.

The experimental configurations are shown in Figure 1. LSG electrodes measuring 2 (cm) in length and featuring either 4 (pt), 1.4 (mm), or 2 (pt), 0.7 (mm) widths were manufactured using an Epilog Mini 12 × 24 60-Watt CO_2_ laser cutter (Epilog Corporation, Golden, CO, USA). The electrodes were inscribed on 5 (mil) thick polyimide film (polyimide Kapton film, DuPont Kapton^®^ HN, Wilmington, DE, USA). The polyimide film was bonded to polyethylene terephthalate (PET) films (0.05 mm and 0.1 mm thickness) and permanent adhesive-backed vinyl sheets (IModeur, Amazon, Seattle, WA, USA) using clear polyester double-sided adhesive tape (ARcare^®^ 90106, Adhesives Research, Glen Rock, PA, USA), with the aim of avoiding temperature-induced deformation of the film and changes in the focus plane during manufacturing. Electrode designs were created in CorelDraw 2021 (Corel Corporation, Ontario, CA, USA) and transferred to the Epilog software (Epilog Job Manager 1.3.6). Electrochemical measurements were performed using a Squidstat Plus potentiostat (Admiral, Austin, TX, USA). Raman spectroscopy measurements were performed using an i-Raman Plus 532S Raman spectrometer (B&W Tek, Newark, DE, USA). The first experimental setup employed a three-electrode system with LSG as the working (W), Pt as the counter (C) electrode (Gamry Instruments, Warminster, PA, USA), and Ag/AgCl as the reference (R) electrode (Gamry Instruments, Warminster, PA, USA). Electrochemical measurements were taken in 10 mL of a 20 mM K_3_Fe[CN]_6_ solution in 0.1 M KCl (Millipore Sigma, Merck KGaA, Darmstadt, Germany) serving as the electrolyte for electrode characterization, Figure 1b. The second experimental setup used two LSG electrodes, manufactured at the optimal settings, as both W and C electrodes, with silver ink (Circuit Writer CW100P silver-based conductive ink, CAIG Laboratories, Poway, CA, USA) as R, Figure 1c,d. In the second set-up, a cover with 2 (mm) diameter holes, and a polymethyl methacrylate (PMMA) rectangular chamber (approximately volume: 200 µL) were used in place of a laboratory flask to reduce and control the deposited electrolyte volume, Figure 1d. The rectangular chamber was fabricated by laser-cutting a 3 mm thick sheet of PMMA (McMaster-Carr, Los Angeles, CA, USA) to obtain the desired geometry for the chamber component.

### 2.1. Electrochemical Characterization

Cyclic voltammetry measurements were conducted using a solution of 20 mM K_3_Fe[CN]_6_ in 0.1 M KCl as the electrolyte. Potential sweeps ranged from −0.4 V to 0.6 V at varying scan rates (10, 20, 30, 40, 50 mV/s) to determine the redox current *I_p_* (A). These measurements facilitated the calculation of current density, *J_p_* (A/m^2^), and the EASA. The Randles–Sevcik equation was used to approximate the EASA as described by Equation (1),

(1) $$I_{p}=26.86{\;\times \;10}^{4}n^{3/2}D^{1/2}v^{1/2}CA_{R-S}$$

where $I_{p}$ (Amperes) is the redox current peak, n is the number of electrons contributing to the redox reaction (n = 1 in this case), D (cm^2^/s) is the diffusion coefficient (7.6 × 10^−6^ cm^2^/s) [40], v (V/s) is the scan rate, C (mol/cm^3^) is the concentration of the probe molecule and $A_{R-S}$ is the estimated EASA using the Randles–Sevcik equation.

The capacitance method using Equation (2) validated the EASA results,

(2) $$A_{c}=\frac{C_{dl}}{C_{s}}$$

where $C_{dl}$ (F) is the double-layer capacitance, estimated from the slope of the capacitive current versus scan rate plot in the non-Faradaic region, $C_{s}$ (F/cm^2^) is the specific capacitance, and $A_{c}$ is the estimated EASA calculated using the capacitance method.

### 2.2. Design of Experiments Using FFD and CCD

The Epilog laser cutter uses five parameters to control the manufacturing process: laser head speed (Sp), laser power (Pw), drawing dots per inch (DPI), the electrode width (Wh) as drawn on CorelDraw, and laser focus. Identifying the most relevant manufacturing parameters was done by measuring the peak redox current (I_p_) and EASA from electrodes made from different manufacturing parameter combinations. Two parameters were maintained constant: the DPI at 1200 DPI and the laser beam on the focus plane of the laser system (the laser was focused manually using the Epilog focusing tool). Experiments were conducted by varying the three manufacturing parameters: laser head speed, laser power, and electrode width to “high” and “low” settings. The values for “high” and “low” for each parameter were chosen based on experience.

**Figure 2:**
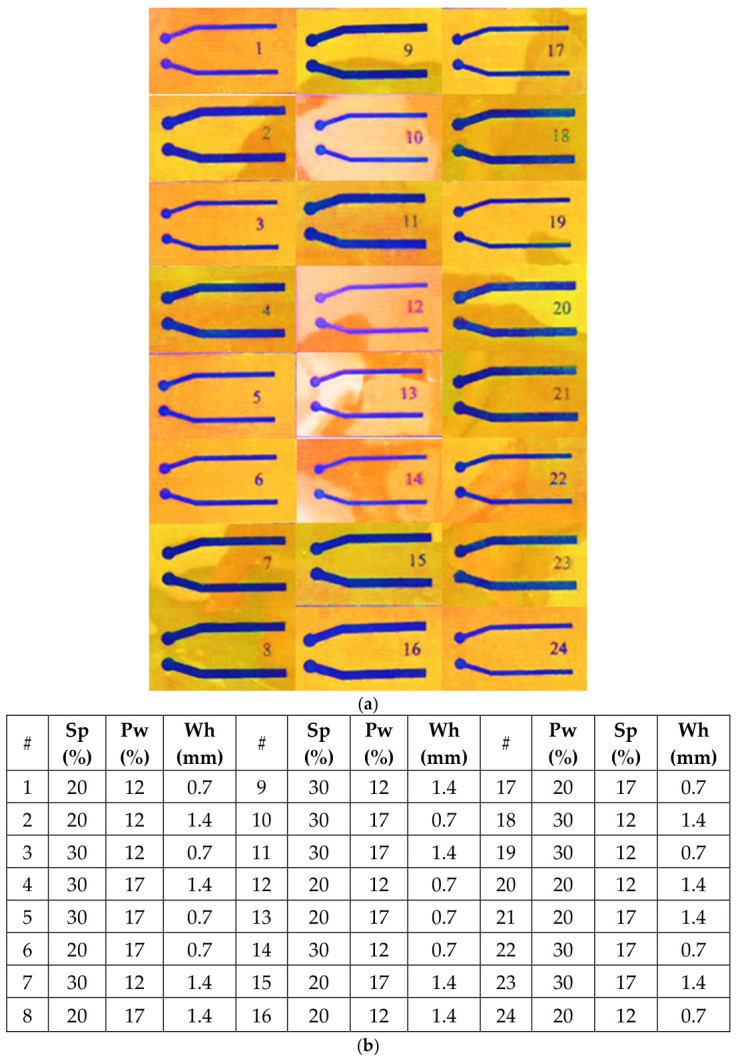
Electrode matrix manufactured using a CO_2_ laser cutter for a 2^3^ full factorial design. (**a**) Electrodes manufactured in random order, using high and low levels for each parameter: Speed (Sp: 20%, 30%), Power (Pw: 12%, 17%), and Width (Wh: 0.7 mm, 1.4 mm). (**b**) Manufacturing parameter matrix showing three replicates for each manufacturing configuration.

A 2^3^ FFD was implemented using Minitab Statistical Software (v20.2, Minitab, LLC, State College, PA, USA) to determine the effect of speed, power, and width for each configuration evaluated. The first configuration used laser head speeds of 20% and 30% of maximum (83.33 mm/s maximum speed, measured experimentally), laser power levels of 12% and 17% of maximum power (60 Watts), and electrode widths of 2 (pt), 0.7 (mm), and 4 (pt), 1.4 (mm), yielding the electrode matrix shown in Figure 2. Electrodes were manufactured in randomly selected locations according to the labeled number, from 1 to 24. One of each pair was used as the working electrode to measure *I_p_* using a scan rate of 10 (mV/s) from −0.4 (V) to 0.6 (V) for the first experimental setup, shown in Figure 1b.

**Figure 3:**
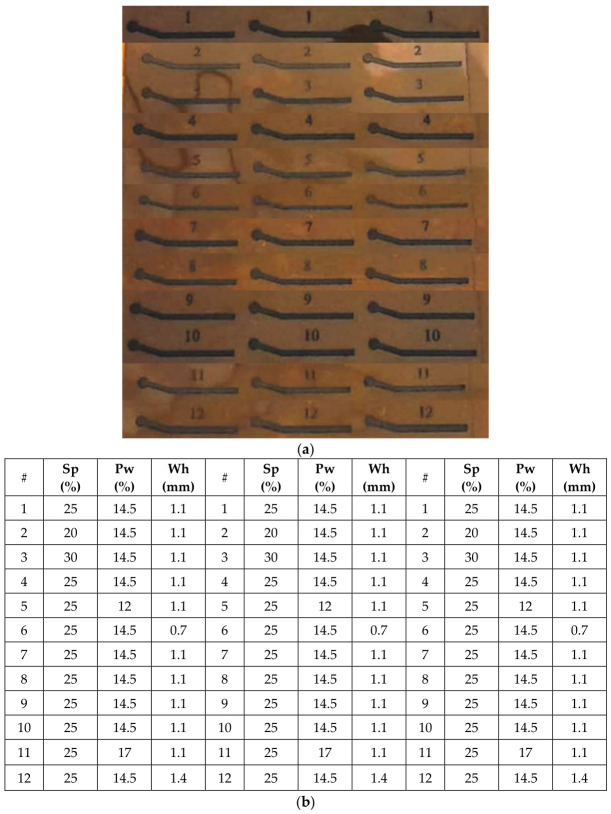
Electrodes used for central composite design (**a**) Electrodes manufactured in random order, using three levels for each parameter: Speed (Sp: 20%, 25%, 30%), Power (Pw: 12%, 14.5%, 17%), and Width (Wh: 0.7 mm, 1.1 mm, 1.4 mm). (**b**) Manufacturing parameter matrix showing three replicates for each manufacturing configuration.

In a second analysis, central regions of the design are explored using CCD, which involves a total of 36 experiments. These experiments consist of six in the face-centered region and six in the central design, with three replicates for each one, as shown in Figure 3.

### 2.3. Raman Measurements

Raman measurements were obtained using an i-Raman Plus 532S Raman spectrometer with a laser of 532 nm and coupled to a microscope using a 20× objective, to focus on the electrodes and reduce the effect of the polyimide. The acquisition conditions were 30 (s) integration time, three scans, and 25 (mWatt) laser power. Spectra were preprocessed with smoothing using Savitzky–Golay with an 11-point window, then normalized with standard normal variate (SNV), and baseline corrected. Z. Xu et al. [41] presented the equation of penetration depth for different wavelengths (d_p = 2.3/2α), where α corresponds to the absorption coefficient (α = 4πk/λ), k is the extinction coefficient, and λ is the wavelength [42]. For graphene and graphite structures, the extinction coefficient has a value of k = 1 and k = 1.3, respectively [43]. Therefore, for the 532 (nm) lasers, the penetration depth of the carbon-based electrodes will be between 37 and 49 (nm).

## 3. Results and Discussion

**Figure 4:**
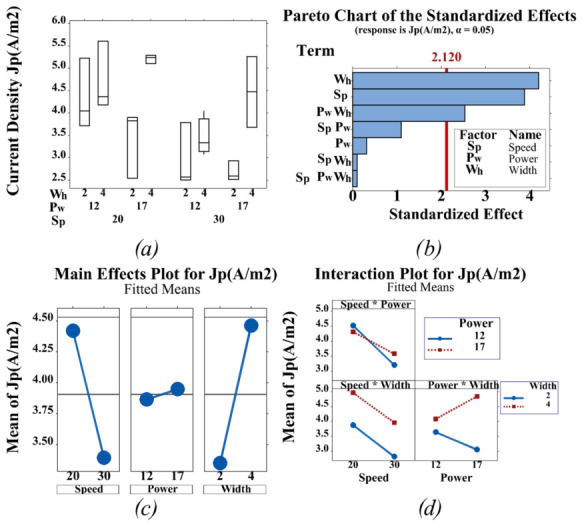
2^3^ Full factorial design results. (**a**) Box plot of current density to identify the changes in each manufacturing parameter combination. (**b**) Pareto chart using a 95% confidence level. (**c**) Main effect for each speed, power, and width. (**d**) Interaction plot for each combination.

Figure 4a shows a box plot illustrating the relationship between the manufacturing parameters and the normalized redox current density, denoted as *J_p_* (A/m^2^). *J_p_* is calculated by dividing *I_p_* by the geometric area of the electrode that is in contact with the electrolyte. The absence of outliers in the data indicates consistent current density under the different manufacturing conditions. Using a 95% confidence level, the Pareto chart in Figure 4b identifies that the most significant manufacturing parameters were the electrode width (Wh), the manufacturing speed (Sp), and the interaction between power and width (Pw-Wh). The relevance of speed, power, and width is further evaluated in Figure 4c, revealing that a high width level and a low-speed level allow a high *J_p_*. Power was not a relevant manufacturing factor for the values used. However, power becomes a relevant parameter when interacting with electrode width, as demonstrated in Figure 4d. Therefore, a preliminary conclusion is that the best configuration corresponds to a high electrode width (4 pt) and low laser head speed (20%). Changing the power levels from 12% to 17% did not have a significant effect. Therefore, laser speed seems to be a more relevant manufacturing parameter than power, at least for the values chosen.

**Figure 5:**
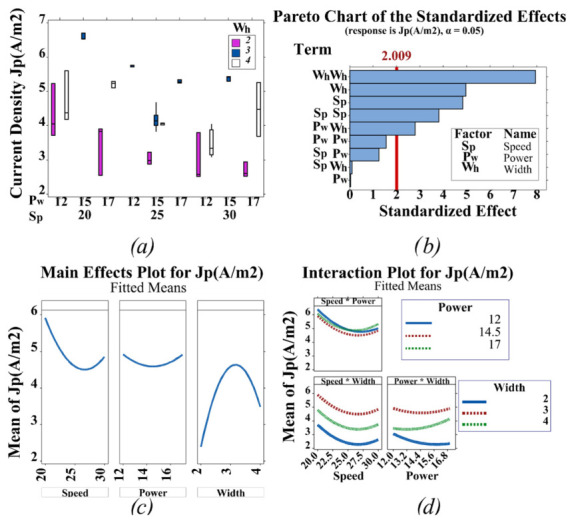
Central composite design results. Sp, Pw, and Wh correspond to Speed, Power, and Width, respectively. Terms such as Sp-Sp, Pw-Pw, and Wh-Wh represent quadratic effects, while Sp-Pw, Pw-Wh, and Sp-Wh represent interaction effects between parameters. (**a**) Box plot for current density to identify the changes in each manufacturing parameter combination. (**b**) Pareto chart using a 95% confidence level. (**c**) Main effect for each speed, power, and width. (**d**) Interaction plot for each combination.

Figure 5a shows a box plot illustrating the relationship between the manufacturing parameters and the measured current density, *J_p_* (A/m^2^). The absence of outliers in the data indicates that the current density remains consistent across the different manufacturing conditions. Using a 95% confidence level, the Pareto chart in Figure 5b again identifies electrode width and laser speed as the most significant factors for electrode manufacturing, validating the FFD analysis. Additionally, laser power and the associated interactions show no statistically significant effects. Figure 5c identifies the main effects of speed, power, and width settings on *J_p_*. The CCD identifies a low-speed setting, 20%, and a medium width, 3 (pt), 1.1 (mm), as the values that achieve the highest *J_p_*. Figure 5d shows the interaction effects between speed, power, and width. Varying the power from 12% to 17% seems to have little effect on *J_p_*. A low speed, 20%, and a width of 3 (pt) achieve the highest *J_p_* for the ranges chosen.

The curvatures in Figure 5c show that the effect of the manufacturing parameters on *J_p_* is nonlinear, suggesting that there are optimum parameters for optimizing electrode performance. In general terms, using the knowledge gained from the CCD and the FFD, we can state that slower and wider are better than faster and thinner, in terms of electrode performance. However, experiments also showed that speeds of 20% or lower resulted in excessive carburization and delamination of the graphene from the substrate, which limited the ability to perform multiple cyclic voltammetry experiments with varying scan rates on a single electrode. Therefore, the next experiment uses constant values of width 4 (pt) and power 12% to further explore the effect of laser head speed on electrode performance.

**Figure 6:**
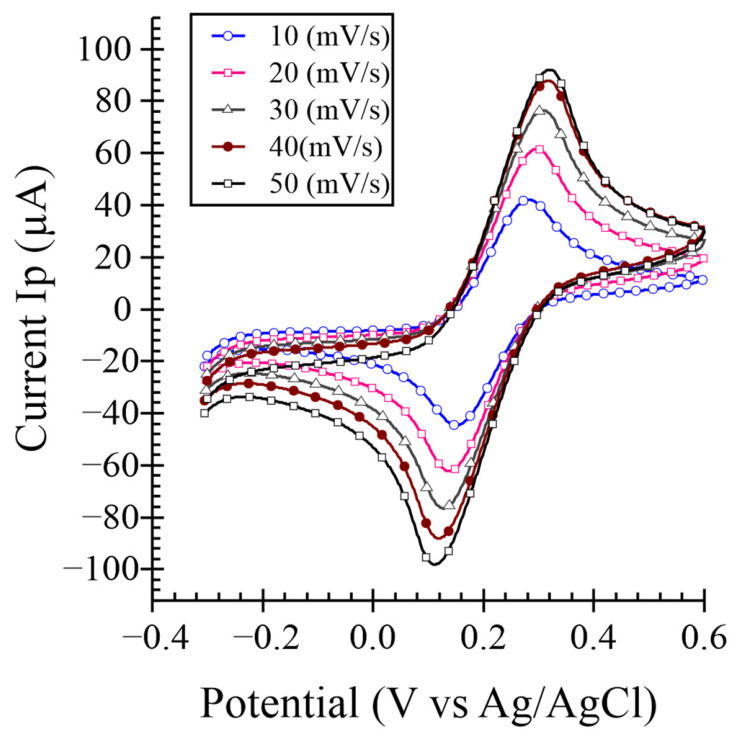
Cyclic voltammetry measurements for LSG electrodes manufactured using 25% speed, 12% power, 4 pt width, and 1200 DPI at voltage rates of 10, 20, 30, 40, 50 (mV/s).

Both FFD and CCD experiments show that laser head speed is the most important laser-related manufacturing parameter for producing electrodes having a high *J_p_*. Consequently, a new set of experiments was conducted to calculate the EASA for electrodes manufactured using different laser speeds (25%, 30%, 35%, and 40%) while maintaining the other parameters constant at power 12% and width 4 (pt). These values were selected based on the earlier analysis, where power was found to be statistically insignificant within the studied range, and a larger width improved electrode performance. Figure 6 shows the cyclic voltammetry results for the 25% speed setting, evaluated at varying voltage scan rates (10, 20, 30, 40, 50 (mV/s)) to characterize the capacitive behavior and the EASA. Although previous results indicated that lower speeds enhance electrochemical performance, speeds below 25% led to excessive carburization and delamination, limiting electrode stability and repeatability. Therefore, a laser speed of 25% was selected as a suitable compromise between achieving high EASA and maintaining structural integrity. The cyclic voltammetry response of the LSG electrodes is based on the reversible redox reaction of K_3_Fe[CN]_6_ solution in KCl. The observed oxidation and reduction peaks correspond to electron transfer processes occurring at the electrode surface. The magnitude of the peak current is governed by the electrochemically active surface area (EASA).

**Figure 7:**
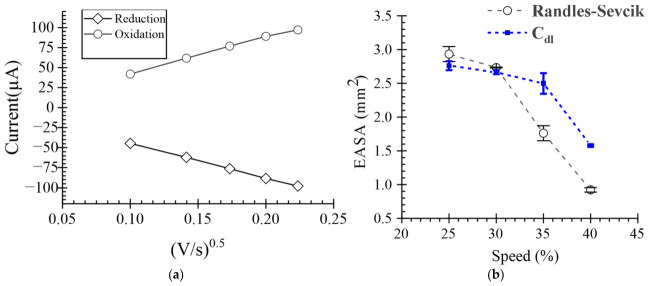
(**a**) Oxidation and reduction peak magnitudes for LSGs manufactured at 25% speed, 12% power, 4 pt width, and 1200 DPI. (**b**) EASA for electrodes made using four different laser head speeds (25%, 30%, 35%, 40%).

The redox current peak magnitudes, Figure 7a, were obtained for each manufacturing speed at each scan rate and used to compute the EASA, following Equation (1). Additionally, the capacitive current, measured in the non-Faradaic region at the same scan rates, was used to determine the double-layer capacitance C_dl_ for each electrode manufactured at different laser speeds. Similarly, the specific capacitance C_s_ was estimated (74.1 µF/cm^2^). These values were applied in Equation (2) to validate the EASA results obtained from Equation (1). Figure 7b shows the EASA as a function of laser speed for both methods described in Equations (1) and (2), showing that the lowest laser speed achieves the highest EASA. The best results were 2.93 ± 0.11 mm^2^ and 2.76 ± 0.07 mm^2^ for Equations (1) and (2), respectively, both obtained at a laser head speed of 25%. This finding aligns with the preliminary conclusion that lower laser speeds increase the redox current *I_p_*, current density *J_p_*, and, in this case, EASA. Given that the electrode width also plays an important role in EASA, a refined preliminary conclusion is to design “wider” electrodes at “slower” laser speeds to achieve a relatively high *I_p_* and EASA, while avoiding excessive carburization and delamination of the material. The definition of “wider” and “slower” will depend on the laser equipment used and the intended application.

To further support the comparison between fabrication conditions, Table 1 summarizes the electrochemically active surface area (EASA) obtained at different laser speeds using both the Randles–Sevcik and capacitance methods. The table includes the corresponding standard deviations, providing a quantitative assessment of electrode performance and reproducibility. As shown, the electrode manufactured at 25% laser speed exhibits the highest EASA values for both methods, confirming the trends observed in Figure 7. This result supports selecting 25% as the optimal fabrication condition, balancing electrochemical performance and structural stability.

**Figure 8:**
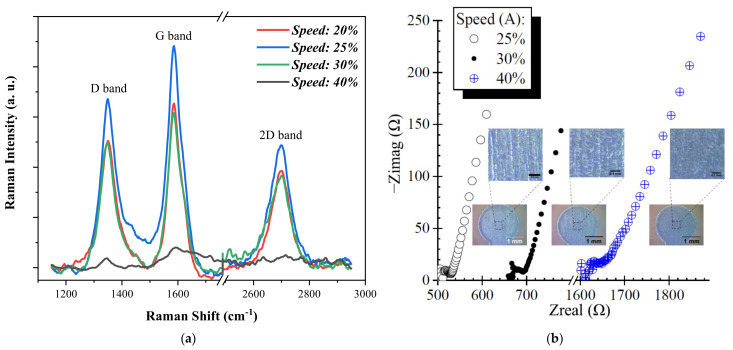
Comparison between structural and electrical properties. (**a**) Raman spectroscopy measurements of electrodes manufactured using power 12% and speed settings 20%, 25%, 30, and 40%. (**b**) Nyquist plot obtained from electrodes made using three different speeds (25%, 30%, 40%), at a power of 12%, and an electrode width of 3 (pt).

Raman spectroscopy experiments were carried out on the LSG electrodes to obtain information on the material properties, disorders, and defects produced in the electrodes by the laser ablation manufacturing process. Figure 8a shows the Raman spectroscopy measurements for electrodes manufactured at 20%, 25%, 30%, and 40% speed, 12% power, 4 (pt) width, 1200 DPI, and a focused laser. The results show that samples manufactured at speeds of 20%, 25%, and 30% present three characteristic peaks of graphene and graphite, known as the D, G, and 2D bands, located around 1349, 1586, and 2700 cm^−1^, respectively. However, the sample manufactured at a speed of 40% shows a reduced intensity of the D and G bands, and the 2D band is not present in the Raman spectrum. This behavior can be attributed to insufficient laser energy input, which limits the effective carbonization of the polyimide substrate and reduces the formation of graphitic (sp^2^) structures. Additionally, the presence of residual polyimide may introduce fluorescence effects, which can mask the Raman signal and hinder the identification of the characteristic D, G, and 2D bands. Fluorescence may also influence the apparent structure of the graphene electrode and, consequently, affect its electrochemical properties. Peaks D and G are characteristic of graphite materials. Peak D is not present in bulk graphite; the detection of this band indicates imperfections in the material, which are associated with defects in carbonaceous systems with sp^2^ hybridization [44,45]. The G band indicates that there is a structure derived from graphite and is associated with the vibrations of the sp^2^ carbon atoms [42]. The 2D band is produced by the second-order resonance of the D band [45]. This band is used as a simple and efficient method to confirm the presence of a single graphene layer [42]. The study by Abdulhafez et al. [46] suggests that the Raman spectra shown indicate the formation of graphene domains in an anisotropic cellular network of 3D graphene. The I_D_/I_G_ ratio for the different speeds (20%, 25%, and 30%) was approximately 0.8, which may indicate that the electrodes present a crystalline structure [45]. The I_2D_/I_G_ ratios were less than 1, suggesting the presence of high defects and disorders in its crystal structure [42], in concordance with Abdulhafez et al. [46]. It is important to note that the laser-scribed graphene (LSG) electrodes consist of a porous, multi-layered network of graphene-like nanocarbon structures rather than a continuous graphene film. Therefore, the Raman intensity ratios (e.g., I_D_/I_G_ and I_2D_/I_G_) should be interpreted qualitatively as indicators of disorder and structural characteristics, rather than as definitive metrics of graphene layer number or crystallinity.

Overall, results suggest that these LSGs are mostly made of multiple graphene-based nanocarbon layers containing a high number of defects and lattice disorders. This can also be somewhat visually perceived by observing the carburized texture of the LSG electrode surface. Interestingly, the electrode manufactured at a 25% speed showed a greater intensity in the D, G, and 2D bands. This electrode also exhibited the lowest *J_p_* in Figure 5c. Structural changes are expected to influence the electrochemical properties of the electrodes. Figure 8b shows electrochemical impedance spectroscopy (EIS) results for the three different laser speeds used. The electrodes measured were made using a constant power of 12% and a width of 3 (pt), while varying the speed (25%, 30%, 40%). Higher speeds were chosen in this experiment to illustrate the trend of increasing Ohmic resistance with higher laser scribing speeds. The electrical resistance of the electrodes is the *x*-axis intercept in the Nyquist plot. Resistance decreases from 1.6 kΩ to 0.52 kΩ while decreasing the laser speed, concurring with the FFD and CCD that lower laser speeds improve electrode performance.

**Figure 9:**
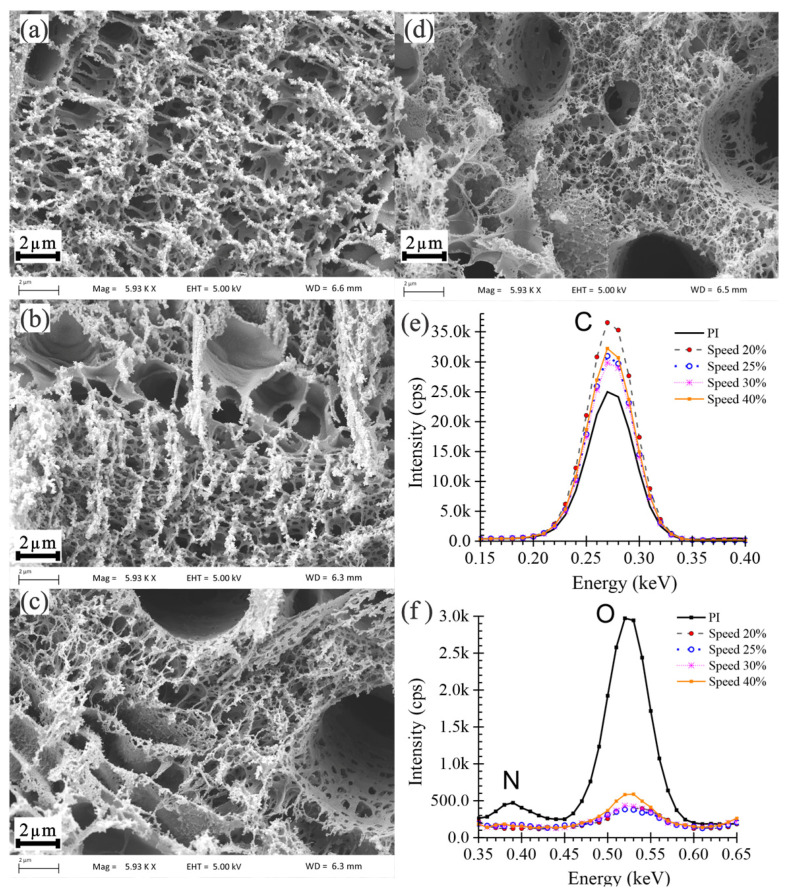
(**a**–**d**) Scanning electron microscopy (SEM) images of LSG electrodes at 5.93 k magnification for laser speed (**a**) 20%, (**b**) 25%, (**c**) 30%, and (**d**) 40%. (**e**,**f**) Elemental characterization of (**f**) carbon, (**e**) nitrogen, and oxygen using energy dispersive spectroscopy (EDS) spectra in the low energy region.

Figure 9a–d shows a structural characterization of laser-scribed graphene (LSG) electrodes using scanning electron microscopy (SEM) images at a magnification of 5.93 kX. The samples used in this characterization were LSG manufactured at a speed of (a) 20%, (b) 25%, (c) 30%, and (d) 40%. The results show a more uniform structure at lower speeds compared with higher speeds. Comparing Figure 9a–d to Figure 7b suggests that the structure created at lower speeds provides the electrode material with a larger electrochemically active surface area (EASA), leading directly to improved conductivity. Figure 9e,f shows the elemental characterization of samples using energy dispersive spectroscopy (EDS) spectra in the lower energy region (0 keV–0.65 keV). Figure 9e shows the characteristic carbon (C) Kα peak at 0.28 keV, with the intensity reflecting the change in carbon content due to different laser scribing speeds. Notably, the lowest manufacturing speed produces a higher carbon concentration compared to polyimide (PI) and the other manufacturing speeds, perhaps also playing an important role in electrode conductivity and EASA. Figure 9f shows the Kα peaks for nitrogen (N) at 0.392 keV and oxygen (O) at 0.525 keV. This region shows a significant drop in O and N signal intensity from the PI to the LSG samples, which confirms the laser-induced reduction and conversion of the polymer to graphene, a key process in electrode fabrication.

**Figure 10:**
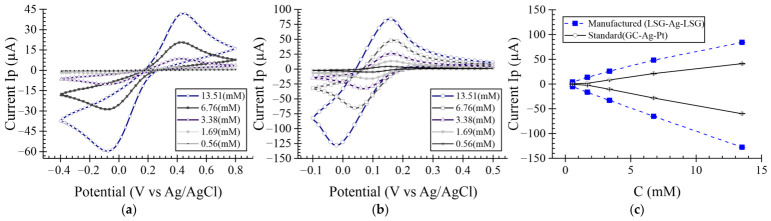
Cyclic voltammetry measurements comparing standard and manufactured electrodes. (**a**) Voltammogram for various molar concentrations using standard electrodes: Pt (counter electrode), Ag/AgCl (reference electrode), and glassy carbon (GC, working electrode). (**b**) Voltammogram for various molar concentrations using manufactured electrodes: LSG (counter electrode), Ag ink (reference electrode), and LSG (working electrode). (**c**) Evaluation of the redox current peak for each concentration, comparing standard and manufactured electrodes.

**Figure 11:**
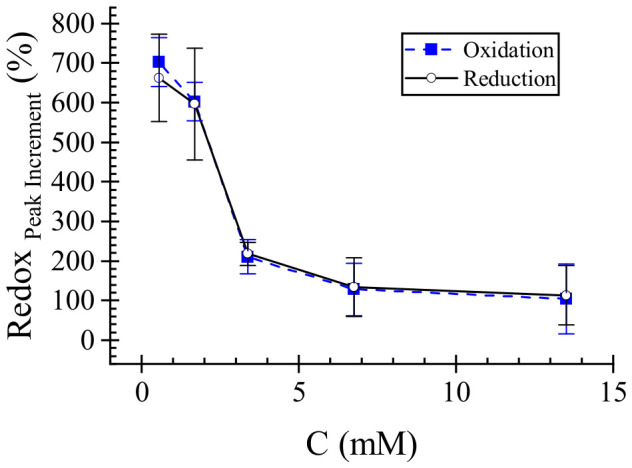
Percentage of signal increment for various molar concentrations using manufactured electrodes (LSG, Ag, LSG) compared to standard electrodes (Pt, Ag/AgCl, GC).

In the subsequent series of experiments, the setup depicted in Figure 1b was used to compare the performance of LSG electrodes with a standard electrode configuration. The manufactured LSG electrodes served as both the working (W) and counter (C) electrodes, while silver ink was employed as the reference (R) electrode. The W and C electrodes were manufactured using 25% speed, 12% power, and a 4 (pt) width, based on the results from Figure 7b and Figure 8b. This electrode arrangement (LSG, Ag ink, LSG) was compared to a standard setup, featuring Pt as C, Ag/AgCl as R, and glassy carbon (GC) as W electrodes. Cyclic voltammetry experiments were conducted using K_3_Fe[CN]_6_ as the electrolyte at five different concentrations (0.56, 1.59, 3.38, 6.76, 13.51 mM) in 0.1 M KCl, with a scan rate of 50 (mV/s). It is important to note that, as expected for high-surface-area, porous carbon-based materials, the LSG electrodes exhibit a characteristic capacitive background current. However, this capacitive contribution does not obscure the Faradaic redox processes, as evidenced by the well-defined anodic and cathodic peaks of the probe. In accordance with benchmarking practices for comparing novel carbon-based electrodes with conventional setups [47], we maintained consistent experimental conditions, allowing us to attribute the observed differences in current response primarily to the material’s intrinsic properties, specifically its enhanced porosity and larger electrochemically active surface area (EASA), rather than non-Faradaic charging effects. The voltammogram obtained with standard electrodes (Pt, Ag/AgCl, GC) is presented in Figure 10a, while Figure 10b shows the voltammogram obtained with manufactured electrodes (LSG, Ag ink, LSG). The results underscore the capability of LSG electrodes to measure various electrolyte concentrations, as evidenced by the increase in *I_p_* with concentration. Notably, LSG electrodes exhibit a more substantial increase in amplitude at each higher electrolyte concentration compared to the standard electrodes. Figure 10c provides a detailed comparison between the two experimental setups through a plot depicting the linear relationship between *I_p_* and concentration. The data from Figure 10c reveal a higher redox current amplitude for LSG compared to GC at each measured concentration. Since the geometric surface area and experimental conditions were kept constant, these differences can be attributed to the intrinsic properties of the electrode materials. The enhanced current response of the LSG electrode is associated with its porous structure and higher electrochemically active surface area (EASA), which was previously characterized through Randles–Sevcik and capacitance methods. The calculated sensitivity, derived from the slope of the linear relationship in Figure 10c for oxidation, is 6.1 µA/mM and 3.2 µA/mM for LSG and glassy carbon, respectively. This steeper slope observed for the LSG electrode’s response indicates a more significant change in current for a given change in concentration, thus suggesting a higher sensitivity than the glassy carbon electrode under the tested conditions. This higher sensitivity is also reflected in the more substantial increase in amplitude observed at higher electrolyte concentrations. Figure 11 displays the percentage increment of the manufactured electrodes compared to the standard electrode at each concentration. The percentage increments are computed according to the following relationship.

(3) $${Redox}_{Peak\;Increment}=\frac{\left|I_{LSG}-I_{GC}\right|}{I_{GC}}\times 100$$

where ${Redox}_{Peak\;Increment}$, is the percentage of the redox peak, $I_{LSG}$, is the redox peak measured with LSG as the working electrode, and $I_{GC}$, is the redox peak measured with GC as the working electrode.

According to Equation (3), the percentage increments in the reduction region are 662%, 596%, 218%, 134%, and 113%, while for the oxidation region are 702%, 602%, 210%, 127%, and 103% for the concentrations used, 0.56, 1.59, 3.38, 6.76, and 13.51 mM, respectively. This suggests that LSG electrodes are particularly suited for detecting low analyte concentrations.

**Figure 12:**
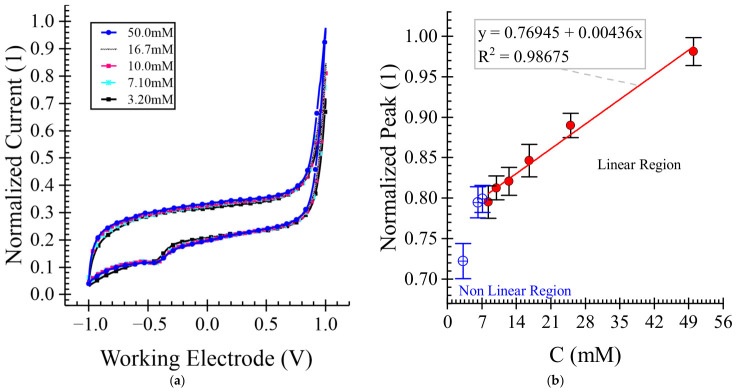
L-Histidine detection in artificial sweat at concentrations ranging from 3.2 mM to 50 mM. (**a**) L-Histidine voltammograms for five representative concentrations (3.2, 7.1, 10, 16.7, 50 mM). (**b**) A linear relationship between L-Histidine concentration and normalized peak current was observed.

Finally, the selected LSG electrode, manufactured with a power of 12%, a speed of 25%, and a width of 4 pt, was employed to obtain voltammograms of artificial sweat for detecting varying concentrations of a single component (L-Histidine) without the need for a biorecognition molecule. These voltammograms were analyzed to investigate how the responses varied as a function of L-Histidine concentration within a range of 3.2 mM to 50 mM. Figure 12a displays voltammograms obtained for varying L-Histidine concentrations at a scan rate of 50 mV/s in a basic sweat buffer solution consisting of a mixture of 0.5% NaCl, 0.05% L-Histidine, and 0.50% Na_2_HPO_4_. Each voltammogram exhibits a significant and irreversible anodic current, which likely corresponds to the one-electron oxidation of the Histidine molecule at the electrode surface, potentially involving interactions with species present at the electrode surface, such as adsorbed oxygen. This irreversibility could be attributed to the subsequent irreversible decarboxylation step, where CO_2_ is released [48]. The oxidation of L-Histidine is expected to occur at electroactive defect sites and edge planes of the LSG electrode, where a higher density of exposed functional groups facilitates charge transfer. The electron transfer process likely involves the interaction of the imidazole group of L-Histidine with these active sites, promoting electron exchange between the molecule and the electrode surface. The porous and defect-rich structure of the LSG enhances this process by increasing the availability of electroactive sites and shortening the electron transfer pathways.

It is important to note that the electrochemical response observed in artificial sweat differs from that obtained using the K_3_Fe[CN]_6_ system due to the higher complexity of the medium, which may introduce interference effects and reduce peak definition. Additionally, the potential window was adjusted to capture the oxidation behavior of L-Histidine, which occurs at different potentials than K_3_Fe[CN]_6_. It should be emphasized that this study primarily focuses on the optimization and fabrication of LSG electrodes, while the detection of L-Histidine in artificial sweat is presented as a proof of concept to demonstrate the applicability of the electrodes in a complex environment. Subsequently, voltage and current data, extracted from the acquired voltammograms, were normalized using maximum-minimum current scaling for analysis. The analysis revealed distinct current peaks for each L-Histidine concentration, as illustrated in Figure 12b. This figure demonstrates a linear relationship (R^2^ = 0.987) between the current peak and L-Histidine concentration within the range of 8.3 mM to 50 mM. Concentrations below 8.3 mM exhibited non-linear behavior. This non-linearity likely arises from the electrochemical kinetics of the analyte at lower concentrations and potential surface adsorption phenomena [49]. A formal limit of detection (LOD) was not determined in this study; however, the results demonstrate a measurable response within the investigated concentration range, supporting the potential of the fabricated electrodes for sensing applications. Detection in this lower, physiologically relevant range could be achieved using more sensitive electrochemical techniques, such as linear sweep voltammetry (LSV), pulse voltammetry (PV), and electrochemical impedance spectroscopy (EIS).

## 4. Conclusions

This study investigated the influence of laser fabrication parameters on the electrochemical and structural properties of laser-scribed graphene (LSG) electrodes. A design of experiments (DoE) approach, combining full factorial design (FFD) and central composite design (CCD), enabled the identification of key factors governing electrode performance. The results demonstrated that laser speed and electrode width play a critical role in enhancing current density (*J_p_*) and electrochemically active surface area (EASA), with lower speeds and wider geometries yielding improved performance within practical fabrication limits.

Structural characterization confirmed that the optimized laser parameters produce a porous, defect-rich graphene-based nanocarbon network, which contributes to enhanced electrochemical activity. The optimized LSG electrodes exhibited strong electrochemical performance and were successfully applied for the detection of L-Histidine in artificial sweat, demonstrating their capability to operate in complex media.

However, several limitations remain. The detection range does not yet fully cover physiologically relevant concentrations, and parameters such as the limit of detection (LOD), selectivity, long-term stability, mechanical flexibility, and reproducibility were not fully evaluated in this study. Additionally, further validation in real biological samples is required to assess practical applicability. Additionally, anti-interference performance against coexisting ions and biomolecules in artificial sweat, as well as validation using real human sweat samples, was not investigated and required further study to assess practical applicability.

Future work will focus on improving sensitivity in the physiologically relevant concentration range, as well as evaluating anti-interference behavior, long-term stability, mechanical robustness under bending conditions, and batch-to-batch reproducibility of the LSG electrodes to advance their use in wearable biosensor applications.

## Figures and Tables

**Table 1:** Summary of electrochemically active surface area (EASA) values obtained for different laser speeds using both the Randles–Sevcik and capacitance methods (mean ± standard deviation). Measurements were performed at constant power (12%) and width (4 pt).

Sp (%)	Pw (%)	Wh (pt)	EASA*_R-S_* (mm^2^)	Stdev	EASA_Cdl_ (mm^2^)	Stdev
25	12	4	2.934	0.112	2.759	0.067
30	12	4	2.734	0.003	2.662	0.031
35	12	4	1.761	0.112	2.498	0.152
40	12	4	0.923	0.035	1.577	0.016

## Data Availability

The data presented in this study are available on request from the corresponding author.
